# Editorial: Sorghum and Pearl Millet as Climate Resilient Crops for Food and Nutrition Security

**DOI:** 10.3389/fpls.2022.851970

**Published:** 2022-03-11

**Authors:** Palak Chaturvedi, Mahalingam Govindaraj, Velu Govindan, Wolfram Weckwerth

**Affiliations:** ^1^Molecular Systems Biology Lab (MOSYS), Department of Functional and Evolutionary Ecology, Faculty of Life Sciences, University of Vienna, Vienna, Austria; ^2^Department HarvestPlus Program, Alliance of Bioversity International and the International Center for Tropical Agriculture (CIAT), Cali, Colombia; ^3^International Maize and Wheat Improvement Center (CIMMYT), Texcoco, Mexico; ^4^Vienna Metabolomics Center (VIME), University of Vienna, Vienna, Austria

**Keywords:** genetic resources, sorghum, pearl millet, climate smart crops, food and nutritional security, finger millet, foxtail millet, breeding

Sorghum and pearl millet serve as a major source of food, feed and fodder for the semi-arid tropical regions of developing world. These two cereal crops rank within the major six cereal crops with a staple food for about 250 million people residing in semi-arid tropic and dryland areas of south Asia and Africa. Sorghum and pearl millet are also regarded as climate-smart crops because of their extreme tolerance to heat (up to 42°C air temperature), drought, and salinity. This Research Topic on Sorghum and Pearl millet but also Finger millet and Foxtail millet as climate resilient and nutrition-rich crops for food and nutrition security comprise 27 manuscripts. It aims to provide new insights into the genetic resources, high throughput precision phenotyping, breeding approaches, multiomics platforms, gene editing, disease resistance, and gene mapping. It also aims to accelerate breeding cycles for climate resilience and improve nutritional quality in these staple cereal crops.

## Sorghum

In the scope of food and nutritional security, understanding wild progenitors of sorghum (Sorghum bicolor) would allow us to exploit the underutilized gene pool to develop more climate-resilient sorghum cultivars. The gene pool of natural sorghum ecotypes may harbor useful gene candidates for both biotic and abiotic stress. Genetic barriers in gene introgression from wild relatives to cultivated sorghum species hold a great challenge. Still, with the recent advent of next-generation sequencing (NGS), more genomic data are available, which expands and extend the sorghum improvement programs using the novel, yet unexploited genes in sorghum's wild relatives (Ananda et al.). Temperature sensitivity and photoperiod of sorghum germplasm are important factors to identify accurate sources for developing cultivars with a broad adaptation, the photoperiod and temperature insensitive, photoperiod and temperature-sensitive and photoperiod sensitive and temperature insensitive sources identified in one of the studies could help breeders to use exact sources in their breeding program, the photoperiod and temperature insensitive accessions can be utilized to develop cultivars with broader adaptation. In contrast, the highly photoperiod sensitive tall accessions can be utilized for biomass and forage improvement and such breeding is suitable in India and USA (Upadhyaya et al.). This segment of research needs more of specific product profile including stress tolerance to meet the special market demand. A major challenge in sorghum breeding is the post-emergence grass weed. 4-hydroxyphenylpyruvate dioxygenase-inhibitor herbicides (e.g., mesotrione or tembotrione) can control a broad spectrum of weeds. The sequencing of 317 sorghum lines and QTL mapping genotypes G-200 and G-350 conferred a very high level of metabolic resistance to tembotrione controlled by a polygenic trait (Pandian et al.). Anthracnose is another devastating fungal biotic stress in sorghum caused by *Colletotrichum saublineola*; a review presented by Abreha and coworkers provides a comprehensive overview of the current knowledge on the mechanisms of sorghum-*C. sublineola* molecular interactions, quantitative trait loci (QTL), and major (R) resistance gene sequences as well as defense-related genes associated with anthracnose resistance (Abreha et al.). A systematic validation of these identified genes and QTLs in coming years can assist in breeding resilient sorghum cultivars for stress prone regions particularly in India and Africa.

The contemporary and updated perspective in understanding the genetic and biochemical interactions between the fungal pathogens, their corresponding mycotoxins, and their host has been reviewed (Ackerman et al.). In a multi-location field study, grain yield (GY) and grain mold resistance was tested. Genotype-by-trait biplots indicated that GY is highly influenced by flowering time, 100-grain weight (HGW), and plant height (PH). In contrast, grain mold resistance was influenced by glume coverage and pH (Aruna et al.). Another important parasitic weed in sub-Saharan Africa is *Striga hermonthica*, it is one of the most devastating factors for sorghum production. To identify new sources of resistance to Striga, in total 64 sorghum genotypes consisting of landraces, wild relatives, improved varieties, and fourth filial generation (F4) progenies were evaluated for both pot and field trail which resulted in more resistant and high-yielding genotypes from F4 derivatives. These genotypes need more acceptance by the farmers (Muchira et al.). Developing drought-tolerant sorghum varieties with high protein content and tolerance to grain mold is highly important. Nagesh et al. identified four sorghum varieties PYPS 2, PYPS 4, PYPS 8, and PYPS 11, which are highly stable in low grain mold incidence (Kumar et al.). This study used additive main effects, multiplicative interaction (AMMI) and genotype × environment interaction (GGE) biplot methods.

Pan-genome analysis of sorghum using reference genomes and 354 genetically diverse sorghum accessions led to the identification of more than two million SNPs; association analysis identified approximately 398 SNPs significantly associated with important agronomic traits. Gene expression analysis under drought identified 1,788 genes that were functionally linked to the cell membrane, catalytic activity, molecular function regulation, response to the stimulus, metabolic process, cellular, and biological regulation. In total, 79 genes were absent from the reference genome assembly (Ruperao et al.). More such research analyses are required to strengthen sorghum pan-genome assembly for increased traits association and its use in breeding program.

Improved Nitrogen Use Efficiency (NUE) is one of the primary goals for the global sorghum improvement programs. Root tissues of contrasting lines exhibited differential expression profiles for transporter genes such as ammonium transporter (SbAMT), nitrate transporters (SbNRT); primary assimilators [glutamine synthetase (SbGS)], glutamate synthase (SbGOGAT[NADH], SbGOGAT[Fd]), assimilatory genes nitrite reductase (SbNiR[NADH]3); and amino acid biosynthesis associated gene [glutamate dehydrogenase (SbGDH)]. Expression profiling of contrasting sorghum genotypes in varying N dosages provides new information in understanding the response of NUE genes toward adaptation to the differential N regimes in sorghum (Bollam et al.). Investigating the biological linkage between and among NUE, stay green and late flowering can offer appropriate breeding road maps for developing optimal NUE in stay green sorghum cultivars in future.

## Pearl Millet

Pearl millet *(Pennisetum glaucum)* breeding in India has historically evolved from open-pollinated varieties to single cross hybrid breeding in a comprehensive manner with closer and continued association of CGIAR and NARS centers. To further accelerate the hybrid breeding efforts for drought-prone areas in South Asia and Sub-Saharan Africa, the heterotic grouping of hybrid parental lines is essential to sustain long-term genetic gains (Yadav O. P. et al.). Pearl millet is nutritionally rich and high in micronutrients such as iron (Fe) and zinc (Zn) and its increased dietary intake can prevent associated hidden hunger or malnutrition. The inclusion of minimum standards for micronutrients such as Fe and Zn content in the cultivar grain release policy is for the first time reported in pearl millet across the globe, motivate institutional commitments and progress toward incorporating essential nutritional traits in breeding pipelines (Satyavathi et al.). QTLs for Fe and Zn content from three distinct production environments were generated using a genetic linkage map consisting of 210 F6 recombinant inbred lines (RIL) population derived from the (PPMI 683 × PPMI 627) cross using genome-wide simple sequence repeats (SSRs). Two constitutive expressing QTLs for Fe and Zn were co-mapped in LG 2. The second one on LG 3, the QTLs candidate genes such as Ferritin gene, Al^3+^, K^+^, Zn^2+^ and Mg^2+^ transporters were identified using bioinformatics approaches (Singhal et al.). In another study, newly developed open-pollinated varieties (30 OPVs of which 8 are Fe/Zn biofortified) were tested for field performance and stability for grain yield, grain Fe and Zn contents across 10 locations in West Africa, resulting in a strong correlation (r = 0.98**) between grain Fe and Zn contents that merit Fe-based selection and can be effective in pearl millet variety breeding (Gangashetty et al.).

Importance of open pollinated varieties cannot be ruled out because of lower input cost, wider adaptation and timely seed availability. OPVs of pearl millet were tested in three different locations across India to check the variation in grain Fe and Zn contents. The results showed a highly significant positive correlation (across environment = 0.83; *p* <0.01), indicating the efficacy of simultaneous selection for both traits (Sanjana Reddy et al.). A set of 105 forage-type hybrid parents of the diverse panel was genotyped following genotyping by sequencing (GBS) and phenotyped for crude protein (CP) under multi-cuts for two consecutive years. This led to the identification of one stable significant single nucleotide polymorphism (SNP) on LG4 for CP. Nine SNPs were distributed across six linkage groups except on LG2 (Govintharaj et al.). These identified loci require validation with robust phenotyping methods in forage gene pool including photo sensitive breeding materials which can facilitate forage quality traits improvement in pearl millet through marker-assisted selection.

Transcript expression profiling for functional classification of a gene belonging to a small heat shock protein (sHSP) family in pearl millet under high-temperature stress led to the identification of two high-temperature-responsive markers Pgcp70 and PgHSF. Physio-biochemical trait screening of the contrasting genotypes among the eight different pearl millet inbred lines at the seedling stage resulted in the identification of PgHSP20 genes, which can provide further insights into the molecular regulation of pearl millet stress tolerance, thereby bridging them together to fight against the unpredicted nature of abiotic stress (Mukesh Sankar, Satyavathi et al.).

Foliar blast disease of pearl millet is severe, caused by *Magnaporthe grisea*. To unravel the G x E interactions for identification and validation of stable resistant genotypes against foliar blast disease through multi-environment testing, a group of 250 different accessions from 20 different countries were collected and screened under natural epiphytotic conditions, which resulted in 43 resistant genotypes which can be used in future resistance breeding programs for pearl millet (Mukesh Sankar, Singh et al.).

Pearl millet accessions that can use nitrogen efficiently needs to be characterized soon. In this aim in total 380 diverse pearl millet lines consisting of a global diversity panel (345), parents of mapping populations (20), and standard checks (15) were evaluated in an alpha-lattice design with two replications. Eleven nitrogen use efficiency (NUE) related traits across three growing seasons in an N-depleted precision field under three different N levels (0%-N0, 50%-N50, 100%-N100 of recommended N, i.e., 100 kg ha^−1^) resulted in 25 top N-tolerant and N-sensitive genotypes under low N conditions. Tolerant genotypes with low N may help identify genomic regions responsible for NUE. Its deployment in pearl millet breeding programs through marker-assisted selection (MAS) can be facilitated (Pujarula et al.). Cabo Verde Islands are poorly explored for genetic resources related to plants. Their potential to supplement the genetic pool of cultivated species is an attempt to identify islands crop wild relatives (CWR) from the Poaceae family and provide a checklist of priority CWR taxa, highlighting particular conservation concerns and the areas which should be the focus of the most intensive conservation efforts in these islands (Rocha et al.). Similarly, the total antioxidant content of pearl millet flour and evaluation of 222 genotypes for antioxidant activity from inbred lines resulted in 18 candidate genes related to antioxidant pathway genes (flavanone 7-O-beta-glycosyltransferase, GDSL esterase/lipase, glutathione S-transferase) residing within or near the association signal that can be selected for further functional characterization (Yadav C. B. et al.).

Multiomics combined with speed breeding is one of the answers to producing highly nutritious food crops (Weckwerth et al., 2020; Yang et al.). Furthermore, integration of the individual omics technique employing the “phenotype to genotype” and “genotype to phenotype” concept together with the systems biology approach may be beneficial for crop breeding improvement under different environmental conditions (Weckwerth et al., 2020). Recently, two important cereal crops, Pearl millet (C_4_) and Wheat (C_3_), were compared at the physiological and proteomics level to understand the drought stress response mechanisms. Tissue-specific proteome analysis of leaves, roots and seeds led to the identification of 12,558 proteins in pearl millet and wheat under well-watered and stress conditions. The physiological response was demonstrated using Odum's model. The study provides for the first time “stay-green” proteomics signatures for Pearl millet (Ghatak et al.). Furthermore, comparative proteome signatures for “stay-green” and “senescence” traits in Pearl millet and wheat under drought stress were identified and correlated with the physiological analysis. NAD-ME type photosynthesis was evaluated in both the cereals, and discriminant analysis via sPLS led to the identification of the putative protein markers, and correlation with an important physiological trait such as root length was determined. This study provides an opportunity to identify important molecular processes in C_4_ traits essential for drought resistance and incorporate them into C_3_ plants via genetic engineering (Ghatak et al.).

## Finger Millet and Foxtail Millet

The Research Topic also consists of manuscripts on finger millet *(Eleusine coracana)* and foxtail millet *(Setaria italica)*, also members of the Poaceae family. Finger millet is an important cereal crop in southern Asia and eastern Africa. It has a long storage period, grows under arid and semi-arid environmental conditions, and has good nutraceutical properties. Blast disease in finger millet caused by the filamentous ascomycetous fungus (*Magnaporthe oryzae*) is the most devastating disease affecting the growth and yield of this crop in all its growing regions. Breeding strategies and challenges in improving this blast disease resistance in finger millet have been extensively reviewed (Mbinda and Masaki). A total of 314 global finger millet germplasm diversity panel accessions were genotyped, using the DArTseq approach to find the genetic diversity and population structure within these genotypes, the authors obtained 33,884 high-quality single nucleotide polymorphism (SNP) markers on 306 accessions after filtering, considerable genetic diversity, and the mean polymorphic information content was determined (Backiyalakshmi et al.). In crops, MADS-box transcription factors play vital roles in multiple biological processes. Genome-wide identification and classification of MADS-box genes in foxtail millet have not been reported previously. In total, 72 MADS-box genes in the foxtail millet genome give an overview of the phylogeny, chromosomal location, gene structures, and potential functions of the proteins encoded by these genes. Expression patterns of 10 foxtail millet MADS-box genes that are upregulated in response to drought were analyzed in different tissues in response to different abiotic stresses because the SiMADS51 genes were found to be strongly induced by drought stress, the function of the SiMADS51 gene was assessed by expression in the model plants Arabidopsis (*Arabidopsis Thaliana*) and rice (*Oryza sativa* L.) (Zhao et al.). In another study, 108 diverse landraces and wild accessions of sorghum, pearl millet, and pigeon pea were studied by genotyping using the DArTSeq approach, which identified 45249 SNPs in pearl millet, 19052 in SNPs sorghum and 8211 SNPs in pigeonpea. Interestingly, sorghum had the lowest average phenotypic (0.090) and genotypic (0.135) variance within accession distances, while pearl millet had the highest average phenotypic (0.227) and genotypic (0.245) distances. These studies are very helpful to the genebank curators to understand the dynamics of the population within accession and support the planning of appropriate germplasm conservation strategies (Allan et al.).

In summary, the variety of studies reported in these diverse crops, pearl millet, sorghum, finger millet and foxtail millet are comprehensive and provides immense knowledge to the coming generations of crop scientists, crop physiologists, plant biologists and breeders. The studies reported in this Research Topic (Volume I) provide us with clear global research goals and are in place on making more climate-resilient crops in future by close observing crop agro-climate variability. Breeders are provided with specific and comprehensive catalogs of important and validated gene candidates that are associated with resilience and nutritional traits. These groundbreaking studies and corresponding breeding programs will eventually enhance crop productivity and improve sorghum and millet-based food intake to meet the food and nutritional security in south Asia and sub-Saharan Africa. They also open up the path to new exploitation of these prestigious cereal crop plants in other regions of the world subject to climate crisis.

## Author Contributions

PC and WW drafted the editorial. MG contributed and provided inputs at the designing stage of the Research Topic. PC, MG, VG, and WW reviewed and revised the editorial. All authors approved the submitted version.

## Conflict of Interest

The authors declare that the research was conducted in the absence of any commercial or financial relationships that could be construed as a potential conflict of interest.

## Publisher's Note

All claims expressed in this article are solely those of the authors and do not necessarily represent those of their affiliated organizations, or those of the publisher, the editors and the reviewers. Any product that may be evaluated in this article, or claim that may be made by its manufacturer, is not guaranteed or endorsed by the publisher.
